# Associations between cerebral amyloid and changes in cognitive function and falls risk in subcortical ischemic vascular cognitive impairment

**DOI:** 10.1186/s12877-017-0522-4

**Published:** 2017-06-28

**Authors:** Elizabeth Dao, John R. Best, Ging-Yuek Robin Hsiung, Vesna Sossi, Claudia Jacova, Roger Tam, Teresa Liu-Ambrose

**Affiliations:** 1 Department of Physical Therapy, University of British Columbia, 212 – 2177 Wesbrook Mall, Vancouver, BC V6T 1Z3 Canada; 2 Djavad Mowafaghian Centre for Brain Health, University of British Columbia, 2215 Wesbrook Mall, Vancouver, BC V6S 0A9 Canada; 3 Department of Medicine, University of British Columbia, UBC Hospital S152, 2211 Wesbrook Mall, Vancouver, BC V6T 2B5 Canada; 4 Department of Physics and Astronomy, University of British Columbia, 6224 Agricultural Road, Vancouver, BC V6T 1Z1 Canada; 5 School of Graduate Psychology, Pacific University, 190 SE 8th Avenue, Hillsboro, OR 97123 USA; 6 MS/MRI Research Group, University of British Columbia, 2215 Wesbrook Mall, Vancouver, BC V6S 0A9 Canada; 7 Centre for Hip Health and Mobility, Robert H.N. Ho Research Centre, 2635 Laurel Street, Vancouver, BC V5Z 1M9 Canada

**Keywords:** Vascular cognitive impairment, Alzheimer’s disease, Amyloid, Cognitive impairment, Executive functions, Falls risk

## Abstract

**Background:**

To determine the association between amyloid-beta (Aβ) plaque deposition and changes in global cognition, executive functions, information processing speed, and falls risk over a 12-month period in older adults with a primary clinical diagnosis of subcortical ischemic vascular cognitive impairment (SIVCI).

**Methods:**

This is a secondary analysis of data acquired from a subset of participants (*N* = 22) who were enrolled in a randomized controlled trial of aerobic exercise (NCT01027858). The subset of individuals completed an ^11^C Pittsburgh compound B (PIB) scan. Cognitive function and falls risk were assessed at baseline, 6-months, and 12-months. Global cognition, executive functions, and information processing speed were measured using: 1) ADAS-Cog; 2) Trail Making Test; 3) Digit Span Test; 4) Stroop Test, and 5) Digit Symbol Substitution Test. Falls risk was measured using the Physiological Profile Assessment. Hierarchical multiple linear regression analyses determined the unique contribution of Aβ on changes in cognitive function and falls risk at 12-months after controlling for experimental group (i.e. aerobic exercise training or usual care control) and baseline performance. To correct for multiple comparisons, we applied the Benjamini-Hochberg procedure to obtain a false discovery rate corrected threshold using alpha = 0.05.

**Results:**

Higher PIB retention was significantly associated with greater decrements in set shifting (Trail Making Test, adjusted R^2^ = 35.3%, *p* = 0.002), attention and conflict resolution (Stroop Test, adjusted R^2^ = 33.4%, *p* = 0.01), and information processing speed (Digit Symbol Substitution Test, adjusted R^2^ = 24.4%, *p* = 0.001) over a 12-month period. Additionally, higher PIB retention was significantly associated with increased falls risk (Physiological Profile Assessment, adjusted R^2^ = 49.1%, *p* = 0.04). PIB retention was not significantly associated with change in ADAS-Cog and Verbal Digit Span Test (*p* > 0.05).

**Conclusions:**

Symptoms associated with SIVCI may be amplified by secondary Aβ pathology.

**Trial registration:**

ClinicalTrials.gov, NCT01027858, December 7, 2009.

## Background

Alzheimer’s disease (AD) and subcortical ischemic vascular cognitive impairment (SIVCI) are the two most common causes of cognitive dysfunction [1], but people often present with mixed pathology [2]. Pathological hallmarks of AD include the presence of amyloid-beta (Aβ) plaques and neurofibrillary tangles (NFT) [3]. On the other hand, SIVCI is characterized by the presence of white matter hyperintensities (WMH) and lacunes. In the last decade, there is growing recognition of the high prevalence of mixed presentations [1, 2, 4] and many studies have begun to investigate the impact of cerebrovascular pathology in AD [5–7]. However, few studies have considered mixed pathology from a primary SIVCI diagnosis perspective.

It is important to consider AD pathology within an SIVCI diagnosis as both share common pathogenic mechanisms – studies indicate a positive feedback loop effect between Aβ and cerebrovascular dysfunction. For example, Aβ may cause vascular dysregulation by compromising cerebral perfusion, reducing vascular reserves, and increasing the propensity for ischemic damage. In return, hypoxia and/or ischemia may promote the production of the Aβ peptide resulting in greater Aβ plaque accumulation [8]. Molecular studies indicate a close interaction between AD and SIVCI pathology [8, 9]; yet, few studies have been conducted to investigate the association between Aβ and cognitive function in SIVCI [10–12].

Furthermore, elevated levels of Aβ have been associated with cognitive dysfunction. Increased Aβ plaque deposition, identified by positron emission tomography (PET), was associated with decreased episodic memory performance and global cognitive function in healthy older adults, mild cognitive impairment (MCI), and AD participants [13, 14]. Though executive dysfunction is a characteristic of early AD [15], few studies have assessed the potential impact of Aβ on executive functions. One published study found no association between high Aβ deposition and executive functions in MCI; however, the assessment of executive functions was limited to a composite of inhibition and verbal fluency [16]. Another study found that baseline global Aβ deposition in frontal, parietal, and medial temporal cortices was associated with greater decreases in executive functions, language, attention, information processing speed, and visuospatial function at 2-year follow-up in people with MCI [17]. Furthermore, several recent studies have reported an association between amyloid and decreased mobility. Specifically, increased amyloid was associated with decreased gait speed in cognitively normal and mildly impaired older adults [18–20]. One study assessing specific gait parameters reported slower gait speed, lower cadence, longer double support time, and greater stance time variability in older adults with high amyloid [20]. Of particular relevance to our study, a 12-month prospective study found that higher Aβ deposition was associated with a faster time to first fall in community-dwelling healthy older adults [21]. These results indicate that Aβ may have an effect on both cognitive and mobility outcomes.

Although previous studies have begun to evaluate the impact of Aβ on cognitive and physical function in healthy older adults, MCI, and AD participants [13, 14], such studies in people with SIVCI are more limited [11, 12, 22, 23]. Furthermore, previous studies lack a comprehensive assessment of executive functions, information processing speed, and to our knowledge, no studies have assessed falls risk [11, 22, 23]. Also, much of current knowledge is based on cross-sectional studies and few studies have assessed the association of cerebral Aβ on changes in cognitive and mobility outcomes. To address these knowledge gaps, we conducted a secondary analysis to assess the association of Aβ with changes in cognitive function (i.e., global cognition, executive functions, and information processing speed) and falls risk over a 12-month period. We hypothesized that elevated Aβ plaque deposition would be associated with larger decrements in these measures over a 12-month period.

## Methods

Ethical approval was obtained from the Vancouver Coastal Health Research Institute (V07–01160) and the University of British Columbia’s Clinical Research Ethics Board (H07–01160). All subjects gave written informed consent in accordance with the Declaration of Helsinki.

### Participants and study design

This was a planned secondary analysis of data acquired from a proof-of-concept randomized controlled trial (RCT) of aerobic exercise in people with SIVCI (NCT01027858) [24]. Briefly, participants were randomized to either a 6-month thrice-weekly aerobic exercise training group or a usual care control group. Participants were followed for an additional 6-months after completing the 6-month intervention period. Assessments for cognitive functions and falls risk were performed at baseline, 6-month, and 12-month time points. To maximize our ability to detect changes in cognitive function and falls risk, we used baseline and 12-month data for our analyses.

Participants were recruited from the University of British Columbia Hospital Clinic for AD and Related Disorders, the Vancouver General Hospital Stroke Prevention Clinic, and specialized geriatric clinics in Metro Vancouver, British Columbia. The diagnosis of SIVCI was confirmed in each participant by a neurologist based on the presence of cerebral small vessel disease and cognitive impairment [25]. A clinical MRI or computed tomography (CT) scan was used to determine the presence of cerebral small vessel disease which was based on the presence of periventricular and deep white matter lesions and at least one lacunar infarct and the absence of non-lacunar territorial (cortical and/or cortico-subcortical) strokes or other specific causes of white matter lesions (i.e. MS, leukodystrophies, sarcoidosis, brain irradiation). Mild cognitive impairment was defined as a Montreal Cognitive Assessment (MOCA) score < 26/30 at baseline [26]. SIVCI diagnosis also required evidence of progressive cognitive decline (compared with previous level of cognitive function) as confirmed through medical records or caregiver/family member interviews. Overall, participants were generally functioning independently and living in the community with minimal assistance by family or caregiver.

Study Inclusion Criteria: Both inclusion and exclusion criteria have been published previously [24]. Briefly, individuals were eligible for study entry if they met the following criteria: 1) aged 55 years or older; 2) MOCA score < 26/30 at screening [26]; 3) Mini-Mental State Examination score (MMSE) ≥ 20 at screening [27]; 4) if participants are on cognitive medications (e.g. donepezil, galantamine, rivastigmine, memantine, etc.) they must be on a stable and fixed dose that is not expected to change during the 12-month study period, or, if they are not on any of these medications, they are not expected to start them during the 12-month study period; and 5) provide written informed consent. Exclusion criteria included: 1) diagnosed with dementia of any type (e.g. Alzheimer’s disease, dementia with lewy bodies, frontal-temporal dementia) or other neurological conditions (e.g. multiple sclerosis, Parkinson’s disease); 2) taking medications that may negatively affect cognitive function; and 3) participation in a clinical drug trial concurrent to this study.

This analysis included a sub-set of 22 participants (exercise group *n* = 11; control group *n* = 11) who met the overall study eligibility criteria and volunteered to complete a PET scan.

### Descriptive variables

At baseline, we collected information regarding age, sex, body mass index, waist-hip ratio, and comorbid conditions were measured using the Functional Comorbidity Index. In addition, we report WMH volume for a subsample of participants.

### Dependent variables

#### Global cognitive function

ADAS-Cog: This test primarily measures memory, language, and praxis. There are 11 items and scores range from 0 to 70 with higher scores indicating greater cognitive dysfunction [28].

#### Executive functions

Trail Making Test (Part B minus A): This test primarily measures set shifting [29]. Participants were asked to draw lines connecting encircled numbers sequentially (Part A) and to alternate between numbers and letters (Part B). The difference in time to complete Part B and Part A was calculated; smaller difference indicated better performance.

Verbal Digit Span Test (Forward minus Backward): This test primarily measures working memory [30]. Participants repeated progressively longer random number sequences in the same order as presented (forward) and in the reversed order (backward). The difference in score between the two tests was calculated; smaller difference indicated better performance.

Stroop Test: This test primarily measures selective attention and conflict resolution [31]. Participants completed three conditions (80 trials each): 1) reading out color words printed in black ink; 2) reading out the display color of colored-X’s; and 3) participants were shown a page with color-words printed in incongruent colored inks and were asked to name the ink color in which the words were printed. The time difference between the third condition and second condition was calculated; smaller difference indicated better performance.

#### Information processing speed

Digit Symbol Substitution Test (DSST): This test primarily measures information processing speed and psychomotor speed [29]. Participants were first presented with a legend of numbers (1 to 9) and their corresponding symbols. They were then presented with a series of numbers, organized in a pre-defined random order, and were asked to fill in the corresponding symbol. Participants were given 90 s to complete the task. A higher number of correct answers in this time period indicated better performance.

#### Falls risk

Physiological Profile Assessment (PPA): This test assesses falls risk [32]. The PPA involves the following subscales: 1) proprioception; 2) edge contrast sensitivity; 3) quadriceps strength; 4) hand reaction time; and 5) postural sway. Each item has a relative weighting and a summary z-score is calculated that indicates: mild risk (0–1); moderate risk (1–2); high risk (2–3); and marked risk for future fall (3 and above) [32]. The PPA is a reliable [33] and valid [34, 35] measure of falls risk in older adults.

### Independent variable

#### Amyloid-β imaging

Details of the Aβ imaging protocol have been published previously [12]. PET scans were performed using ^11^C–Pittsburgh Compound-B (PIB) produced at UBC TRIUMF. Scans were performed in 3-D mode using the GE Advance tomograph (General Electric, Canada/USA). A 90-min dynamic acquisition started at tracer injection and data were framed into a 18 × 300 sec imaging sequence.

Parametric images of the non-displaceable binding potential (BP_ND_) [36] were generated using tissue input Logan graphical analysis [37, 38] with the cerebellum as the reference region [39, 40]. A mean PIB-PET image was created by averaging radiotracer concentration over the entire scan duration – this image was used for co-registration and ROI definition purposes. Using SPM 8 (Wellcome Department of Cognitive Neurology, Institute of Neurology, University College London) each subject’s T1-weighted MRI image was co-registered to the corresponding mean PIB-PET image. Each subject’s MRI image was then normalized to the SPM MNI305 template and the corresponding transformation parameters were applied to the subject’s PET images (mean and parametric images). For those without MRI scans (5 subjects did not scan due to MR contraindications), the subject’s mean PIB-PET image was normalized to an average PIB-PET image template of 6 healthy control participants.

Regions of interest (ROIs) analysis: A custom set of ROIs was defined on the coronal view of the MNI305 template [41]. These ROIs were transposed to each subject’s warped MRI and mean-PET images (in MNI space) and adjusted as necessary. The modified set of ROIs was then applied to the parametric PIB-PET image and the average PIB BP_ND_ within each ROI was extracted. Global PIB binding was determined by averaging values in bilateral frontal (combined orbitofrontal and medial prefrontal cortex), parietal (combined angular gyrus, superior parietal, precuneus, and supramarginal gyrus), temporal (combined lateral temporal and middle temporal gyrus), and occipital cortices, striatum (putamen and caudate nucleus), and anterior and posterior cingulate gyrus [10].

### Statistical analysis

All statistical analyses were performed using Statistical Package for the Social Sciences 22.0. We conducted a hierarchical multiple linear regression to determine the unique contribution of Aβ plaque deposition on 12-month change in cognitive function and falls risk. We controlled for experimental group (i.e. aerobic exercise training or usual care control) and baseline score. Age was initially included as a covariate but it did not significantly alter the results and was removed for a parsimonious model. The dependent variable for all models was change in the outcome of interest. Change in ADAS-Cog, Trail Making Test, Verbal Digit Span Test, Stroop Test, and PPA was calculated as baseline minus 12-month scores. Change in DSST was calculated as12-month minus baseline scores. In all instances, higher change scores represent improved performance. We report adjusted *R* ^2^ values, which penalizes the explained variance for each additional covariate, resulting in a more realistic estimate of explained variance. For each hierarchical regression model, we computed collinearity statistics (tolerance and variance inflation factor), histograms of the residuals, and scatterplots of the predicted versus residual values to ensure that the assumptions of linear regression were met. In all models, mutlicollinearity was not an issue among predictor variables, and the residuals were normally distributed and homoscedastic. To correct for multiple comparisons across all regression models, we applied the Benjamini-Hochberg [42] procedure to obtain a false discovery rate (FDR) corrected threshold using alpha = 0.05.

## Results

### Descriptive variables

The mean age was 72 ± 7.91 years (minimum age = 56 years; maximum age = 84 years), the average MOCA score was 23.32 ± 2.08, and global PIB BP_ND_ was 0.10 ± 0.23. Six out of 22 participants did not complete an MRI scan – 5 people had MR contraindications and 1 MRI scan was discarded due to severe motion artifacts. Among the 16 participants with MRI data, WMH volume ranged from 76.38–10,058.89 mm^3^ with an average of 2004.40 ± 2761.15 mm^3^. Compared with the participants in the RCT that did not complete PIB scans, this subset was similar in age (mean difference = 3.80, *p* > 0.05), but had a higher mean MOCA score (mean difference = 3.14, *p* ≤ 0.05). Detailed demographic characteristics and neuropsychological test results are presented in Table 1.

**Table 1:** Descriptive Characteristics

Variable	Exercise group *n* = 11		Control group *n* = 11		Total *N* = 22	
	Mean	SD	Mean	SD	Mean	SD
Age	70.00	7.29	73.45	8.47	71.73	7.91
Female sex, No. (%)	3	27.3	4	36.4	7	31.81
MOCA	22.73	2.20	23.91	1.87	23.32	2.08
Waist-to-hip ratio	0.90	0.08	0.93	0.08	0.91	0.08
Body mass index	27.14	6.24	26.75	2.98	26.95	4.78
FCI	3.09	1.81	3.55	1.69	3.32	1.73
Total medications	3.27	3.10	5.00	3.90	4.14	3.55
Beta-blockers, No. (%)	3	27	2	18	5	23
Global PIB BP_ND_	0.14	0.24	0.07	0.23	0.10	0.23
WMH volume (mm^3^)	1277.82^a^	1446.90	2569.52^b^	3450.16	2004.40^c^	2761.15
Baseline Assessments						
ADAS-Cog	10.65	4.76	8.61	2.60	9.63	3.88
TMT B-A, sec.	45.07	20.93	55.95	28.14	50.51	24.84
VDST F-B, sec.	2.45	2.88	4.00	2.61	3.23	2.79
Stroop CW-C, sec.	68.33	30.26	54.54	20.02	61.44	26.01
DSST	26.27	8.01	25.27	5.97	25.77	6.91
PPA	0.54	1.50	0.47	0.91	0.50	1.21
Final Assessments						
ADAS-Cog	9.57	4.70	6.86	2.77	8.22	4.01
TMT B-A, sec.	76.43	69.32	66.45	51.88	71.44	59.97
VDST F-B, sec.	2.18	1.83	3.27	1.85	2.73	1.88
Stroop CW-C, sec.	66.41	25.91	55.60	32.37	61.00	29.14
DSST	26.09	8.93	23.55	8.47	24.82	8.59
PPA	0.12	1.23	0.54	0.59	0.33	0.96

*SD* Standard Deviation, *MOCA* Montreal Cognitive Assessment (max. Score 30), *FCI* Functional comorbidity index (total number of comorbidities), *ADAS-Cog* Alzheimer’s Disease Assessment Scale - Cognitive subscale (max. Score of 70), *TMT B-A* Trail Making Test, Part B minus Part A, *VDST F-B* Verbal Digit Span Test, Forward minus Backward, *Stroop CW-W* Stroop Color Words minus Stroop colored x’s, *DSST* Digit Symbol Substitution Test, *PPA* Physiological Profile Assessment; ^a^Exercise group - WMH volume, *n* = 7; ^b^Control group - WMH volume, *n* = 9; ^c^Total - WMH volume, *n* = 16

### Global cognitive function

ADAS-Cog: PIB BP_ND_ was not significantly associated with change in ADAS-Cog (*p* > 0.05).

### Executive functions

Trail Making Test (Part B minus A): Higher PIB BP_ND_ was significantly associated with decreased set shifting (β = −0.68, *p* < 0.01), the total adjusted variance accounted by the final model was 38.5% – Table 2.

**Table 2:** Multiple regression model assessing the contribution of amyloid on change in Trail Making Test

Independent variables	R^2^	Adjusted R^2^	R^2^ change	Unstandardized B (Standard error)	Standardized β	*P*- value
Step 1	0.04	−0.07	0.04			0.70
Group				20.16 (25.44)	0.18	0.44
TMT baseline				0.07 (0.52)	0.03	0.90
Step 2	0.47	0.39	0.44^a^			<0.01
Group				5.42 (19.70)	0.05	0.79
TMT baseline				0.40 (0.41)	0.18	0.34
PIB BP_ND_				−166.43 (43.09)	−0.68	<0.01

*TMT* Trail Making Test, Part B minus Part A baseline score

^a^Significant after FDR adjustment

Verbal Digit Span Test (Forward minus Backward*)*: PIB BP_ND_ was not significantly associated with change in working memory (*p* > 0.05).

Stroop Test: Higher PIB BP_ND_ was significantly associated with decreased selective attention and conflict resolution (β = −0.54, *p* = 0.01), the total adjusted variance accounted by the final model was 31.4% – Table 3.

**Table 3:** Multiple regression model assessing the contribution of amyloid on change in Stroop Test

Independent variables	R^2^	Adjusted R^2^	R^2^ change	Unstandardized B (Standard error)	Standardized β	*P*- value
Step 1	0.13	0.04	0.13			0.26
Group				2.12 (11.12)	0.04	0.85
Stroop baseline				0.37 (0.22)	0.38	0.11
Step 2	0.41	0.31	0.28^a^			0.01
Group				−0.74 (9.46)	−0.02	0.94
Stroop baseline				0.45 (0.19)	0.46	0.03
PIB BP_ND_				−59.67 (20.46)	−0.54	0.01

*Stroop CW-W* Stroop Color Words minus Stroop colored x’s baseline score

^a^Significant after FDR adjustment

### Information processing speed

DSST: Higher PIB BP_ND_ was significantly associated with decreased information processing speed (β = −0.56, *p* = 0.01), the total adjusted variance accounted by the final model was 20.6% – Table 4.

**Table 4:** Multiple regression model assessing the contribution of amyloid on change in Digit Symbol Substitution Test

Independent variables	R^2^	Adjusted R^2^	R^2^ change	Unstandardized B (Standard error)	Standardized β	*P*- value
Step 1	0.02	−0.08	0.02			0.79
Group				−1.55 (2.26)	−0.16	0.50
DSST baseline				−0.01 (0.17)	−0.01	0.96
Step 2	0.32	0.21	0.30^a^			0.01
Group				−2.45 (1.97)	−0.25	0.23
DSST baseline				−0.09 (0.15)	−0.12	0.56
PIB BP_ND_				−12.31 (4.41)	−0.56	0.01

*DSST* Digit Symbol Substitution Test baseline score

^a^Significant after FDR adjustment

### Falls risk

PPA: Increased PIB BP_ND_ was significantly associated with increased falls risk (β = −0.39, *p* = 0.03), the total adjusted variance accounted by the final model was 51.3% – Table 5.

**Table 5:** Multiple regression model assessing the contribution of amyloid on change in Physiological Profile Assessment

Independent variables	R^2^	Adjusted R^2^	R^2^ change	Unstandardized B (Standard error)	Standardized β	*P*- value
Step 1	0.45	0.39	0.45			<0.01
Group				−0.45 (0.32)	−0.24	0.17
PPA baseline				0.49 (0.13)	0.62	<0.01
Step 2	0.58	0.51	0.13^a^			0.03
Group				−0.56 (0.29)	−0.30	0.07
PPA baseline				0.39 (0.13)	0.50	0.01
PIB BP_ND_				−1.60 (0.67)	−0.39	0.03

*PPA* Physiological Profile Assessment baseline score

^a^Significant after FDR adjustment

## Discussion

Currently, much of our knowledge on the effects of co-existing Aβ pathology in SIVCI is based on cross-sectional studies [11, 12, 22] and little is known about their impact on changes in cognitive function and mobility over time. We found that higher Aβ deposition was associated with greater decrements in set shifting, attention and conflict resolution , and information processing speed over a 12-month period. In addition, we found that people with greater Aβ deposition displayed increased falls risk at follow-up. These results indicate that co-existing Aβ plaque deposition may play a role in subsequent cognitive and mobility declines in older adults with SIVCI.

Previous studies assessing the role of Aβ on cognitive function in SIVCI have produced equivocal results. One study found that Aβ was correlated with decreased performance on tests of immediate and delayed recall of verbal learning but not executive functions [22]. Another study found that Aβ was independently associated with cognitive impairment in multiple domains, including language, visuospatial, memory, and executive functions [11]. A published cross-sectional analysis of this data set found that increased Aβ plaque deposition was associated with poorer performance in global cognitive function as measured by ADAS-Cog [12]. In the current analysis, we did not find an association between Aβ plaque deposition and change in global cognitive function; however, we found that greater Aβ deposition was associated with declines in specific executive processes and information processing speed over 12-months.

Our findings suggest that symptoms associated with SIVCI (i.e. executive dysfunction and decreased information processing speed) are amplified by secondary Aβ pathology. Few studies have been conducted to assess the impact of Aβ on change in cognitive function in people with a clinical diagnosis of SIVCI. The Amyloid PET Imaging for Subcortical Vascular Dementia (AMPETIS) study found that PIB positivity was associated with faster declines in attention, visuospatial skills, visual memory, episodic memory, and verbal learning, but no significant declines in executive functions were detected [23]. However, we note that only a single executive measure was included – phonemic and semantic verbal fluency. Although verbal fluency tests do involve aspects of executive control, they do not isolate the three main components of executive functions: set shifting, working memory/updating, and inhibition of dominant responses [43] – which we targeted in the present study. Furthermore, we report that greater Aβ plaque deposition is associated with reduced information processing speed over time. In summary, these preliminary analyses indicated that co-existing Aβ plaques may be detrimental to multiple domains of cognitive function in people with SIVCI.

We also found that increased Aβ plaque deposition was associated with increased falls risk. This concurs with previous literature – a study in healthy community-dwelling older adults found that higher Aβ was associated with faster time to first fall over 12-months [21]. In addition, epidemiological studies found that 42% of a community sample with mild to moderately severe AD fell within a 12-month period [44, 45]. This is supported by studies that have identified impaired static and dynamic balance, mobility, and gait dysfunction in early AD [45], which may contribute to an increased risk of falling. Furthermore, there is a strong association between cognition, particularly executive functions, with gait and balance. Older people with poor executive control walk slower, have increased stride variability, have poorer performance on complex mobility tasks, and fall more often [46]. Executive dysfunction may impair planning, control, and execution of movements, and thus, can increase falls risk [47].

Our findings are not without limitations. First, this study was a secondary analysis of an exercise intervention trial and it is unclear how exercise may have influenced cognitive function and falls risk. To minimize exercise effects, we statistically controlled for group membership. Second, our small sample size requires that these findings be confirmed in larger follow-up studies. Third, we did not control for the presence of other AD and SIVCI pathologies such as NFT, lacunes, or WMH. This is important to note, as NFT have been associated with cognitive outcomes in AD. Lacunes and WMH have been associated with both executive dysfunctions and falls risk. However, the presence of Aβ has also been associated with executive functions and falls risk in people with MCI and AD, indicating a unique contribution of Aβ on cognitive and mobility declines. Within a subset of this data with WMH volume quantification, including WMH volume and age as covariates did not significantly alter the results. As such, it is plausible that Aβ plaque deposition may independently contribute to changes in cognitive function and falls risk in SIVCI.

## Conclusions

The results of this study suggest that cerebral Aβ plaque deposition is associated with greater declines in both executive functions and information processing speed, as well as greater increases in falls risk among older adults with a primary SIVCI diagnosis. However, more studies with larger samples and longer follow-up are needed to fully elucidate the impact of co-existing Aβ on disease progression in SIVCI. Future therapies for SIVCI may need to account for the potential presence and effect of amyloid for the optimal care of those with SIVCI.

## Abbreviations

AD: Alzheimer’s disease
Aβ: Amyloid-beta
BP_ND_: Non-displaceable binding potential
CT: Computed tomography
DSST: Digit Symbol Substitution Test
FDR: False discovery rate
MCI: Mild cognitive impairment
NFT: Neurofibrillary tangles
PET: Positron emission tomography
PIB: ^11^C Pittsburgh compound B
PPA: Physiological Profile Assessment
RCT: Randomized controlled trial
ROI: Regions of interest
SIVCI: Subcortical ischemic vascular cognitive impairment
WMH: White matter hyperintensities
